# Anemia, iron, and HIV: decoding the interconnected pathways: A review

**DOI:** 10.1097/MD.0000000000036937

**Published:** 2024-01-12

**Authors:** Emmanuel Ifeanyi Obeagu, Getrude Uzoma Obeagu, Nkiruka Rose Ukibe, Samson Adewale Oyebadejo

**Affiliations:** a Department of Medical Laboratory Science, Kampala International University, Ishaka, Uganda; b School of Nursing Science, Kampala International University, Ishaka, Uganda; c Department of Medical Laboratory Science, Nnamdi Azikiwe University, Nnewi, Anambra State, Nigeria; d Department of Biomedical Laboratory Sciences, Faculty of Fundamental Applied Sciences, Institut d’ Enseignement Superiuor de Ruhengeri (INES-RUHENGERI), Musanze District, Northern Region, Rwanda.

**Keywords:** anemia, antiretroviral therapy, inflammation, iron and HIV

## Abstract

This review delves into the intricate relationship between anemia, iron metabolism, and human immunodeficiency virus (HIV), aiming to unravel the interconnected pathways that contribute to the complex interplay between these 3 entities. A systematic exploration of relevant literature was conducted, encompassing studies examining the association between anemia, iron status, and HIV infection. Both clinical and preclinical investigations were analyzed to elucidate the underlying mechanisms linking these components. Chronic inflammation, a hallmark of HIV infection, disrupts iron homeostasis, impacting erythropoiesis and contributing to anemia. Direct viral effects on bone marrow function further compound red blood cell deficiencies. Antiretroviral therapy, while essential for managing HIV, introduces potential complications, including medication-induced anemia. Dysregulation of iron levels in different tissues adds complexity to the intricate network of interactions. Effective management of anemia in HIV necessitates a multifaceted approach. Optimization of antiretroviral therapy, treatment of opportunistic infections, and targeted nutritional interventions, including iron supplementation, are integral components. However, challenges persist in understanding the specific molecular mechanisms governing these interconnected pathways. Decoding the interconnected pathways of anemia, iron metabolism, and HIV is imperative for enhancing the holistic care of individuals with HIV/AIDS. A nuanced understanding of these relationships will inform the development of more precise interventions, optimizing the management of anemia in this population. Future research endeavors should focus on elucidating the intricate molecular mechanisms, paving the way for innovative therapeutic strategies in the context of HIV-associated anemia.

## 1. Introduction

Anemia, characterized by a deficiency in red blood cells or hemoglobin, represents a significant health concern globally.^[1]^ Its prevalence is notably pronounced in individuals living with human immunodeficiency virus (HIV) infection, where the interplay between anemia, iron metabolism, and the viral pathogenesis creates a complex and dynamic web of interactions.^[2]^ This intricate relationship poses both clinical and therapeutic challenges, necessitating a comprehensive exploration to decipher the interconnected pathways that govern this triad.

Anemia is a well-documented complication in the course of HIV infection, contributing to increased morbidity and mortality.^[3]^ The etiology of anemia in HIV is multifaceted, involving direct viral effects, opportunistic infections, medication side effects, and the overarching influence of chronic inflammation.^[4]^ As individuals with HIV are now living longer due to advances in antiretroviral therapy (ART), the management of anemia has become increasingly crucial for improving overall health outcomes.^[5]^ Iron, a vital micronutrient, plays a pivotal role in numerous physiological processes, including oxygen transport, energy production, and immune function.^[6]^ In the context of HIV, disturbances in iron metabolism are not only a consequence of the viral infection but also contribute to the pathogenesis of anemia.^[7]^ Chronic inflammation induced by HIV disrupts the delicate balance of iron homeostasis, influencing erythropoiesis and exacerbating anemia.^[8–12]^

Understanding the interconnected pathways between anemia, iron metabolism, and HIV is essential for the development of targeted and effective therapeutic strategies. While the relationship between these components is recognized, the specific molecular mechanisms governing their interplay remain elusive. Unraveling these intricate pathways holds promise for refining treatment approaches and addressing anemia in the context of HIV more effectively. This review aims to provide a comprehensive exploration of the interconnected pathways linking anemia, iron metabolism, and HIV. By synthesizing existing knowledge, we seek to elucidate the underlying mechanisms, identify potential therapeutic targets, and highlight areas for future research. Through this endeavor, we aim to contribute to the growing body of knowledge that informs clinical practice and enhances the care of individuals living with HIV-associated anemia.

## 2. Methodology

Different reputable databases like PubMed/MEDLINE, Embase, Web of Science, Scopus, and the Cochrane Library were utilized in writing this paper considering anemia, iron and HIV as keywords for the literature searches.

## 3. Anemia in HIV patients

Anemia represents a pervasive and intricate complication in individuals living with HIV. Its presence not only significantly impacts the quality of life for those affected but also poses challenges for healthcare providers managing the multifaceted nature of HIV infection.^[13–17]^ Anemia is a prevalent comorbidity in the HIV population, with estimates suggesting a higher incidence compared to the general population.^[18]^ The prevalence varies at different stages of HIV infection, with a notable increase as the disease progresses.^[19]^ Despite advancements in ART, anemia remains a persistent concern, affecting both adults and pediatric patients living with HIV.^[20]^

The etiology of anemia in HIV is multifactorial, encompassing both HIV-related and non-HIV-related factors.^[21]^ Direct viral effects on hematopoiesis, opportunistic infections such as mycobacterium avium complex and cytomegalovirus, medication side effects (particularly zidovudine), and the chronic inflammatory state induced by HIV contribute to the development and exacerbation of anemia.^[22]^ Additionally, coexisting conditions such as nutritional deficiencies and coinfections further complicate the landscape. Anemia in HIV patients is associated with a range of clinical implications, including increased morbidity and mortality.^[23]^ It correlates with accelerated disease progression, higher rates of opportunistic infections, and a decline in overall immune function. Fatigue, weakness, and reduced exercise tolerance are common symptoms, impacting the daily lives of individuals living with HIV.^[24]^

The diagnosis of anemia in HIV patients involves a comprehensive assessment, including a complete blood count, iron studies, and, if necessary, bone marrow examination.^[25]^ Regular monitoring is essential due to the dynamic nature of both HIV infection and anemia.^[26]^ Identifying the specific etiology is crucial for tailored management strategies. Effective management of anemia in HIV patients requires a holistic approach.^[27]^ Optimization of ART, treatment of opportunistic infections, and correction of nutritional deficiencies, including iron supplementation, are essential components.^[28]^ Erythropoiesis-stimulating agents may be considered in certain cases, and blood transfusions might be necessary in severe instances.^[29]^

Ongoing research endeavors focus on unraveling the nuanced mechanisms of anemia in the context of HIV and exploring novel therapeutic interventions.^[30]^ Tailoring treatments to individual patient profiles and addressing the broader socio-economic determinants of health will be pivotal in improving outcomes for individuals grappling with both HIV and anemia.^[31]^ Anemia in HIV patients is a multifaceted challenge, necessitating a comprehensive understanding of its underlying causes and tailored therapeutic interventions.^[32]^ As advancements in HIV care continue, addressing anemia will play a crucial role in enhancing the overall health and well-being of individuals living with this complex viral infection.

## 4. Iron metabolism in anemia and HIV

Iron, an essential micronutrient, is a linchpin in various physiological processes, including oxygen transport, DNA synthesis, and immune function.^[33]^ The intricate relationship between iron metabolism, anemia, and HIV infection unveils a complex interplay that extends beyond mere deficiency.^[34]^ This paper explores the disruptions in iron homeostasis, their contribution to anemia, and the nuanced impact of HIV on iron metabolism. Iron metabolism is a tightly regulated process involving absorption in the gastrointestinal tract, transportation in the blood bound to transferrin, and storage in the liver as ferritin. Erythropoiesis, a major consumer of iron, depends on this delicate equilibrium.^[35]^ Any perturbation can lead to either iron overload or deficiency, each carrying distinct implications for health.^[36]^

Anemia, characterized by a reduction in red blood cell mass, often has iron dysregulation at its core.^[37]^ In iron-deficiency anemia, insufficient iron impairs the synthesis of hemoglobin, while in anemia of chronic disease, a common occurrence in chronic infections, including HIV, iron is sequestered in macrophages, limiting its availability for erythropoiesis.^[38]^ HIV infection introduces additional layers of complexity to iron metabolism.^[39]^ Chronic inflammation, a hallmark of HIV, triggers hepcidin release, an iron-regulatory hormone.^[40–47]^ Elevated hepcidin levels lead to decreased iron absorption and sequestration, contributing to the anemia observed in HIV patients.^[48]^ Furthermore, the virus itself may modulate cellular iron transporters, influencing iron distribution within the body.

In the context of HIV, anemia is not solely a consequence of impaired iron availability.^[49]^ Viral effects on bone marrow, opportunistic infections, and adverse effects of ART contribute significantly.^[50]^ Disturbances in iron metabolism emerge as part of the intricate web of interactions, exacerbating anemia and influencing disease progression. Understanding the nuances of iron metabolism in HIV-associated anemia is critical for diagnosis and management. Traditional iron markers may not accurately reflect the dynamic shifts in iron distribution. Careful consideration of hepcidin levels, ferritin, and soluble transferrin receptor may provide a more comprehensive assessment of iron status in HIV patients.^[51]^ Managing anemia in HIV requires a tailored approach that addresses both iron dysregulation and other contributing factors.^[52]^ Optimization of ART, treatment of opportunistic infections, and targeted iron supplementation are key components.^[53]^ However, the delicate balance between correcting iron deficiency and preventing iron overload requires careful consideration.

The interconnected pathways of iron metabolism, anemia, and HIV create a dynamic landscape where disruptions in one element reverberate across the others.^[54]^ Unraveling the complexities of this interplay is crucial for devising effective therapeutic strategies that not only address anemia but also contribute to the comprehensive care of individuals living with HIV. As research advances, a deeper understanding of these relationships will pave the way for more precise and nuanced interventions.

## 5. Impact of anemia on HIV progression

Anemia, a common hematological complication in individuals living with HIV, goes beyond the confines of red blood cell deficits.^[55]^ This paper explores the multifaceted impact of anemia on the progression of HIV, emphasizing the intricate interplay between hematological compromise and the dynamics of viral infection. Anemia emerges as a significant predictor of accelerated HIV disease progression. Diminished oxygen-carrying capacity compromises cellular and tissue oxygenation, exacerbating the toll on an already compromised immune system. This synergistic effect creates a challenging milieu, fostering an environment conducive to viral replication and disease advancement.

Anemia’s impact extends beyond its physiological constraints, influencing the very immune system tasked with combatting HIV.^[56]^ Impaired erythropoiesis, a hallmark of anemia, contributes to the depletion of CD4+ T cells, a crucial component of the immune response.^[57]^ This dual assault on both erythrocytes and immune cells sets the stage for a vicious cycle, amplifying the challenges of managing HIV.^[58]^ Individuals with anemia in the context of HIV are more susceptible to opportunistic infections.^[59]^ The compromised oxygenation of tissues weakens the body’s defense mechanisms, creating an opportune environment for the proliferation of pathogens. Opportunistic infections further strain the immune system, hastening the progression of HIV.^[60]^

The effectiveness of ART, a cornerstone in HIV management, is influenced by the presence of anemia.^[61]^ Reduced oxygen delivery compromises the bioavailability of medications, potentially diminishing their therapeutic efficacy. Additionally, the hematological side effects of certain antiretroviral drugs may exacerbate anemia, necessitating careful consideration in treatment planning. Anemia significantly diminishes the quality of life for individuals living with HIV.^[62]^ Fatigue, weakness, and reduced exercise tolerance, common manifestations of anemia, contribute to a decreased overall well-being. Furthermore, the symptomatic burden of anemia can impact medication adherence, posing an additional challenge in the management of HIV.

Anemia serves as an ominous prognostic marker in the HIV landscape.^[63]^ Its presence heralds an increased risk of mortality and complications, emphasizing the need for vigilant monitoring and targeted interventions to mitigate its impact on disease progression. The impact of anemia on HIV progression extends beyond the conventional boundaries of hematological compromise.^[64]^ It intertwines with immune dysfunction, opportunistic infections, and the efficacy of therapeutic interventions, shaping the trajectory of HIV disease.^[65]^ Recognizing and addressing anemia in the holistic care of individuals living with HIV is paramount, not only for hematological correction but for the broader goal of optimizing outcomes and enhancing the overall well-being of those navigating the complexities of HIV infection.^[66]^

## 6. Potential mechanisms linking anemia, iron, and HIV

Central to the interconnected pathways linking anemia, iron dysregulation, and HIV is chronic inflammation.^[67]^ HIV infection induces a persistent inflammatory state characterized by elevated cytokines, including interleukin-6.^[68]^ This inflammatory milieu stimulates hepcidin production, a key regulator of iron homeostasis.^[69]^ Hepcidin, in turn, inhibits intestinal iron absorption and promotes sequestration of iron in macrophages, contributing to anemia by limiting iron availability for erythropoiesis.^[70]^ HIV exhibits direct effects on bone marrow function, impacting erythropoiesis and leading to a decrease in red blood cell production.^[71]^ The virus may infect hematopoietic progenitor cells and interfere with the normal differentiation and maturation processes, contributing to anemia.^[72]^

ART, while indispensable for managing HIV, can introduce an additional layer of complexity.^[73]^ Certain antiretroviral drugs, such as zidovudine, may cause bone marrow suppression, leading to reduced red blood cell production and exacerbating anemia.^[74–75]^ Careful consideration of the choice and management of ART is crucial in addressing both HIV and anemia concurrently. HIV infection can lead to altered iron distribution in different tissues.^[65]^ Some studies suggest increased iron levels in the liver and macrophages, contributing to iron sequestration and limiting its availability for erythropoiesis.^[76,77]^ Simultaneously, iron deficiency in other tissues may occur, creating a paradoxical scenario where iron is both elevated and deficient in various compartments.^[78]^

Opportunistic infections common in HIV, such as mycobacterium avium complex and cytomegalovirus, further complicate the relationship between anemia, iron, and HIV.^[79]^ These infections can directly impact erythropoiesis and contribute to anemia, creating a synergistic effect with HIV-induced mechanisms. HIV-associated immune activation plays a role in disturbing erythropoiesis.^[80]^ Dysregulated cytokine production and immune activation contribute to the suppression of erythropoietin, a hormone essential for red blood cell production.^[81]^ The resulting imbalance in erythropoiesis further contributes to the development of anemia in individuals living with HIV.^[82]^

HIV infection induces oxidative stress, creating a redox imbalance that affects red blood cell survival and function. Oxidative damage to red blood cells can lead to hemolysis and contribute to the development of anemia.^[83]^ Additionally, oxidative stress may impact iron metabolism and exacerbate the inflammatory response. HIV-related gastrointestinal dysfunction can disrupt iron absorption, contributing to iron deficiency anemia.^[84]^ Malabsorption, coupled with chronic inflammation, amplifies the risk of anemia by limiting the availability of iron for erythropoiesis.

The intricate web of potential mechanisms linking anemia, iron dysregulation, and HIV involves a dynamic interplay of viral effects, inflammatory responses, medication influences, and coexisting infections.^[85]^ Unraveling these pathways is essential for developing targeted therapeutic interventions that address the complexity of anemia in the context of HIV, offering new avenues for improved patient care and outcomes.

## 7. Treatment approaches for anemia in HIV patients

Effective HIV management is foundational in addressing anemia.^[86]^ Optimizing ART, guided by considerations of viral suppression, immune reconstitution, and minimizing medication-induced side effects, can positively impact anemia.^[87]^ Switching to alternative antiretroviral drugs with a lower risk of hematologic complications may be considered when appropriate. Prompt identification and treatment of opportunistic infections are crucial. Opportunistic infections not only contribute to anemia but also exacerbate the immune system’s compromise in individuals living with HIV.^[88]^ Aggressive management of these infections can help alleviate anemia and improve overall health outcomes.

In cases of iron deficiency anemia, iron supplementation may be warranted.^[89]^ However, the decision to administer iron should be made cautiously, considering the potential for iron overload, especially in the presence of chronic inflammation. Oral iron supplements or intravenous iron may be utilized based on individual patient characteristics and response. Erythropoiesis-stimulating agents, such as erythropoietin, can be considered in certain cases of anemia, particularly when there is evidence of impaired erythropoiesis. However, the use of ESAs should be judicious, considering potential risks such as thrombosis and concerns about promoting HIV replication. Close monitoring of hemoglobin levels and patient response is essential.^[90]^

In severe cases of anemia with acute complications or symptoms, blood transfusions may be necessary to rapidly correct the deficit.^[91]^ Transfusion support can provide immediate relief while other therapeutic interventions take effect. However, the decision to transfuse should be individualized, considering the risks and benefits. Addressing nutritional deficiencies is integral to managing anemia in HIV patients. A balanced diet rich in iron, vitamin B12, and folate can contribute to erythropoiesis and overall health.^[92]^ Nutritional counseling and supplementation may be beneficial, especially in individuals with malabsorption or dietary challenges.

Given the role of chronic inflammation in anemia of chronic disease, strategies to manage inflammation can indirectly impact anemia.^[93]^ Anti-inflammatory agents, such as nonsteroidal anti-inflammatory drugs, corticosteroids, or other immunomodulatory therapies, may be considered under specific circumstances. However, the potential risks and benefits must be carefully weighed. Anemia management in HIV requires a holistic and multidisciplinary approach.^[94]^ Regular monitoring of hemoglobin levels, iron studies, and markers of inflammation is essential. Collaborative care involving hematologists, infectious disease specialists, and nutritionists ensures a comprehensive and tailored approach to individual patient needs.

The treatment landscape for anemia in HIV patients is nuanced and multifaceted, reflecting the complexity of the interconnected pathways involved. Individualized care, considering the specific etiology of anemia, coexisting conditions, and the broader context of HIV management, is paramount.^[95]^ Ongoing research to refine therapeutic strategies and explore novel interventions remains crucial in improving outcomes for individuals navigating the intricate dynamics of anemia and HIV.

## 8. Challenges and future research directions

The intricate interplay between anemia, iron metabolism, and HIV presents a formidable challenge.^[96]^ Understanding the specific molecular mechanisms governing these pathways is complicated by their dynamic and multifaceted nature. The paradoxical scenario of both elevated and deficient iron levels in different tissues adds complexity. Deciphering the factors influencing tissue-specific iron distribution and its implications for erythropoiesis remains challenging. ART, while indispensable for managing HIV, can introduce complications such as medication-induced anemia.^[97]^ Balancing the benefits of viral suppression with potential hematological side effects poses a clinical conundrum. Iron supplementation, a common therapeutic approach for anemia, carries the risk of iron overload, especially in the presence of chronic inflammation. Balancing the correction of iron deficiency with the potential for exacerbating inflammatory processes poses a clinical dilemma. Despite advances in understanding the interconnected pathways, therapeutic targets for specifically addressing anemia in the context of HIV remain limited.^[98]^ Developing targeted interventions that can modulate these pathways without compromising overall health is a significant challenge.

In-depth exploration of the molecular mechanisms linking anemia, iron metabolism, and HIV is essential.^[99]^ Identifying key signaling pathways, cellular interactions, and genetic factors will provide a foundation for targeted therapeutic interventions. Further research into the tissue-specific regulation of iron distribution is warranted. Understanding how HIV influences iron levels in different tissues and the impact on erythropoiesis will refine our approach to managing anemia. Advancements in understanding the heterogeneity of anemia in HIV patients can pave the way for personalized treatment strategies. Tailoring interventions based on individual patient profiles, including genetic factors and the specific etiology of anemia, holds promise. Exploring novel therapeutic targets beyond traditional approaches is crucial. Targeting specific molecules involved in the interconnected pathways may unveil innovative strategies for managing anemia in the context of HIV. Longitudinal studies and cohort analyses that track changes in anemia, iron status, and HIV progression over time can provide valuable insights.^[100]^ Understanding the temporal dynamics of these interconnected pathways is essential for developing timely and effective interventions. Integration of big data and systems biology approaches can offer a holistic understanding of the intricate relationships between anemia, iron metabolism, and HIV. Analyzing large datasets may reveal patterns and interactions that are not apparent in smaller-scale studies. Recognizing the impact of socioeconomic determinants on the prevalence and management of anemia in HIV patients is crucial. Future research should explore the social and economic factors influencing healthcare access, nutritional status, and overall health outcomes.

In addressing these challenges and pursuing future research directions, the scientific community can advance our understanding of the interconnected pathways, ultimately leading to more effective interventions for individuals navigating the complex intersection of anemia, iron metabolism, and HIV.

## 9. Implications for clinical and health policy making

Understanding the interconnected pathways between anemia, iron metabolism, and HIV has significant implications for clinical practice and health policy-making:

### 9.1. Clinical implications

#### 9.1.1. Improved patient management.

Understanding how HIV affects iron metabolism and contributes to anemia can aid in better management of anemia in HIV-positive individuals. It can guide healthcare providers in tailoring treatment strategies, including iron supplementation or erythropoietin-stimulating agents, to address anemia effectively while considering HIV-related complications.

#### 9.1.2. Enhanced monitoring.

Knowledge of the interplay between anemia, iron, and HIV can lead to improved monitoring of patients. Regular assessments of hemoglobin levels, iron status, and related parameters can help in early detection and management of anemia and associated complications in HIV-infected individuals.

#### 9.1.3. Optimized treatment strategies.

Insights into these interconnected pathways can assist in optimizing ART regimens. Some ART medications may influence iron metabolism or exacerbate anemia, and understanding these interactions can guide clinicians in selecting appropriate ART combinations.

#### 9.1.4. Preventive interventions.

Identification of modifiable factors influencing anemia in HIV could facilitate preventive interventions. This includes addressing nutritional deficiencies, iron supplementation, or implementing strategies to minimize chronic inflammation, which might contribute to anemia.

### 9.2. Health policy implications

#### 9.2.1. Guideline development.

Evidence-based knowledge on the relationship between anemia, iron, and HIV can contribute to the development or modification of clinical guidelines. These guidelines can help standardize screening, diagnosis, and treatment protocols for anemia in HIV-positive populations.

#### 9.2.2. Resource allocation.

Health policies may need to allocate resources for comprehensive screening programs, ensuring access to iron status assessments and appropriate treatments for anemic individuals living with HIV. This could involve improving access to iron supplements, hematological tests, and healthcare services in affected regions.

#### 9.2.3. Training and education.

Health policies can prioritize education and training programs for healthcare professionals to enhance their understanding of the interconnected pathways. This can result in more informed clinical decisions, improved patient care, and better outcomes for individuals living with HIV and anemia.

#### 9.2.4. Research funding.

Government and funding agencies may prioritize research funding into elucidating further complexities of these interconnected pathways. Investing in research could lead to innovative treatments and interventions for managing anemia in HIV-infected individuals, ultimately improving health outcomes.

Comprehending the intertwined relationships between anemia, iron metabolism, and HIV has far-reaching implications. It can lead to more precise clinical management strategies, inform policy decisions, and potentially improve the overall health outcomes and quality of life for individuals affected by HIV and anemia.

## 10. Conclusion

In unraveling the complex interplay between anemia, iron metabolism, and HIV, it becomes evident that these interconnected pathways create a dynamic and intricate landscape with profound implications for patient health.

Decoding the interconnected pathways of anemia, iron metabolism, and HIV is a continuous and evolving journey. As research progresses, the intricate tapestry of factors contributing to this triad becomes clearer, offering hope for more targeted and effective interventions. Through a multidisciplinary approach that integrates clinical insights with cutting-edge research, the healthcare community stands poised to enhance the quality of life for individuals navigating the complexities of anemia and HIV.

## Author contributions

**Conceptualization:** Emmanuel Ifeanyi Obeagu.

**Methodology:** Emmanuel Ifeanyi Obeagu, Getrude Uzoma Obeagu.

**Supervision:** Emmanuel Ifeanyi Obeagu.

**Validation:** Emmanuel Ifeanyi Obeagu.

**Visualization:** Emmanuel Ifeanyi Obeagu.

**Writing – original draft:** Emmanuel Ifeanyi Obeagu, Getrude Uzoma Obeagu, Nkiruka Rose Ukibe, Samson Adewale Oyebadejo.

**Writing – review & editing:** Emmanuel Ifeanyi Obeagu, Getrude Uzoma Obeagu, Nkiruka Rose Ukibe, Samson Adewale Oyebadejo.
